# Clinical and Laboratorial Features That May Differentiate 46,XY DSD due to Partial Androgen Insensitivity and 5**α**-Reductase Type 2 Deficiency

**DOI:** 10.1155/2012/964876

**Published:** 2011-12-12

**Authors:** Nélio Neves Veiga-Junior, Pedro Augusto Rodrigues Medaets, Reginaldo José Petroli, Flávia Leme Calais, Maricilda Palandi de Mello, Carla Cristina Telles de Sousa Castro, Guilherme Guaragna-Filho, Letícia Espósito Sewaybricker, Antonia Paula Marques-de-Faria, Andréa Trevas Maciel-Guerra, Gil Guerra-Junior

**Affiliations:** ^1^Interdisciplinary Group for the Study of Sex Determination and Differentiation (GIEDDS), Faculty of Medical Sciences (FCM), State University of Campinas (UNICAMP), 13083-970 Campinas, SP, Brazil; ^2^Pediatric Endocrinology Unit, Department of Pediatrics, Faculty of Medical Sciences (FCM), State University of Campinas (UNICAMP), 13083-970 Campinas, SP, Brazil; ^3^Center of Molecular Biology and Genetic Engineering (CBMEG), State University of Campinas (UNICAMP), 13083-875 Campinas, SP, Brazil; ^4^Department of Medical Genetics, Faculty of Medical Sciences (FCM), State University of Campinas (UNICAMP), 13083-970 Campinas, SP, Brazil

## Abstract

The aim of this study was to search for clinical and laboratorial data in 46,XY patients with ambiguous genitalia (AG) and normal testosterone (T) synthesis that could help to distinguish partial androgen insensitivity syndrome (PAIS) from 5**α**-reductase type 2 deficiency (5**α**-RD2) and from cases without molecular defects in the *AR* and *SRD5A2* genes. Fifty-eight patients (51 families) were included. Age at first evaluation, weight and height at birth, consanguinity, familial recurrence, severity of AG, penile length, LH, FSH, T, dihydrotestosterone (DHT), Δ4-androstenedione (Δ4), and T/DHT and T/Δ4 ratios were evaluated. The *AR* and *SRD5A2* genes were sequenced in all cases. There were 9 cases (7 families) of 5**α**-RD2, 10 cases (5 families) of PAIS, and 39 patients had normal molecular analysis of *SRD5A2* and *AR* genes. Age at first evaluation, birth weight and height, and T/DHT ratio were lower in the undetermined group, while penile length was higher in this group. Consanguinity was more frequent and severity of AG was higher in 5**α**-RD2 patients. Familial recurrence was more frequent in PAIS patients. Birth weight and height, consanguinity, familial recurrence, severity of AG, penile length, and T/DHT ratio may help the investigation of 46,XY patients with AG and normal T synthesis.

## 1. Introduction

The disorders of sex development (DSD) with sex ambiguity and 46,XY karyotype can be classified in three main groups: (1) disorders of gonadal development (ovotesticular DSD and partial gonadal dysgenesis), (2) disorders of testosterone synthesis (testosterone biosynthesis defects like steroidogenic acute regulatory protein (STAR) deficiency, side-chain cleavage (CYP11A1) deficiency, 3*β*-hydroxysteroid dehydrogenase type II (HSD3B2) deficiency, 17*α*-hydroxylase/17,20-lyase (CYP17A1) deficiency, 17*β*-hydroxysteroid dehydrogenase III (HSD17B3) deficiency, P450 oxidoreductase (POR) defect; cytochrome b5 (CYB5) defect, and defects in luteinizing hormone action (LHCGR defect)), and (3) disorders of testosterone action [partial (PAIS) androgen insensitivity syndromes] or metabolization (5*α*-reductase type 2 deficiency) [1].

The main diagnosis for patients with ambiguous genitalia and 46,XY karyotype (46,XY DSD) with normal testosterone secretion and normal Müllerian duct regression is PAIS or 5*α*-reductase type 2 deficiency. Before puberty, the phenotypes of 46,XY DSD due to androgen insensitivity syndromes or 5*α*-reductase type 2 deficiency are, in general, indistinguishable, particularly when there is no parental consanguinity (5*α*-reductase type 2 deficiency is an autosomal recessive disorder) or family history consistent with X-linked inheritance (androgen insensitivity syndromes) [2–5].

Despite the multiple genetic causes of 46,XY DSD, around 30–40% of cases remain without diagnosis [6]. Currently, there is a frequent, nongenetic variant of 46,XY DSD characterized by reduced prenatal growth and lack of clear evidence for any associated malformation or steroidogenic defect. Additionally, other studies in undetermined 46,XY DSD report that around 30% of cases are associated with low birth weight, indicating that adverse events in early pregnancy are frequent causes of congenital nongenetic 46,XY DSD [7, 8].

For that reason, the aim of this study was to search for clinical and laboratorial features of 46,XY patients with ambiguous genitalia and normal testosterone synthesis that could help to distinguish PAIS from 5*α*-reductase type 2 deficiency and from cases without molecular defects in the *AR *and *SRD5A2 *genes.

## 2. Methods

In the last 10 years (from January 2001 to December 2010), the Interdisciplinary Group for the Study of Sex Determination and Differentiation (GIEDDS) from the Clinical Hospital of the Faculty of Medical Sciences of State University of Campinas (UNICAMP), Brazil evaluated 58 patients (51 families) with ambiguous genitalia having a 46,XY karyotype and normal testosterone secretion after hCG stimulation. This study was performed according to the Helsinki declaration and was approved by the Ethical Research Committee of Faculty of Medical Sciences of UNICAMP. Informed consent was obtained from all participants and from parents of participants under 18 years of age.

All patients included in this study were born at term. They underwent laparoscopy during genitoplasty, orchidopexy, and/or gonadectomy, and no Müllerian structures were found.

Data were obtained regarding age at first evaluation, weight and length at birth, history of parental consanguinity, family history of ambiguous genitalia or infertility, severity of the ambiguous genitalia (according to Sinnecker et al. classification [9] and Ahmed et al. external masculinization score (EMS) [10]), penile length (in z score according to Gabrich et al. [11]), levels of LH, FSH, total testosterone (T), dihydrotestosterone (DHT), T/DHT ratio, Δ4-androstenedione, and T/Δ4 ratio. LH, FSH, and Δ4-androstenedione were evaluated by chemiluminescence immunoassay, T and DHT by radioimmunoassay. T was evaluated at basal levels in all patients, and in all prepubertal patients, a stimulation test was carried out by giving 1,500 IU of hCG by intramuscular injections for three consecutive days on an outpatient basis. Venous samples were taken before the test and approximately 24 hours after the third hCG injection and T was considered normal if above than 1,5 ng/mL [11]. In patients with complete puberty (Tanner 4 or 5), a hCG-stimulation test was carried out only if T was below 9 ng/mL.

Molecular analyses of *SRD5A2* and *AR* genes were performed for all patients. The eight exons of *AR* gene and the five exons of *SRD5A2* were amplified from genomic DNA using the polymerase chain reaction (PCR) followed by sequencing the fragments with Big Dye Terminator Cycle Sequencing Kit V3.1 Ready Reaction (ABI PRISM/PE Biosystems). The sequences were compared with the normal sequence of each gene (ENSEMBL: ENSG00000049319 and ENSG00000169083) using CLC Sequence Viewer v.6.2 (free software).

According to clinical and molecular data, the cases were classified in three groups of diagnosis: (1) 5*α*-reductase type 2 deficiency, (2) PAIS, and (3) undetermined (without molecular defects in either *AR *or *SRD5A2* gene).

Data were processed in the SPSS program for Windows, version 16.0, and descriptive analyses for continuous variables were made by calculating range, means and standard deviation. The data were compared among the three groups using Chi-square test or Fisher's exact test for categorical variables and Kruskal-Wallis test for continuous variables. For all analyses, a significance level of *P* < 0.05 was adopted.

## 3. Results

Data from all 58 patients are shown in Tables 1, 2, and 3. Nine patients (7 families) showed homozygous or compound heterozygous mutations in *SRD5A2* gene (Table 1), confirming the diagnosis of 5*α*-reductase type 2 deficiency in these patients. Only c.278delG mutation (patient 4: Table 1) was not yet described in the literature. Ten patients (5 families) showed hemizygous mutation in *AR* gene, confirming the diagnosis of PAIS (Table 2). The remaining 39 cases showed normal molecular analysis of *SRD5A2 *and *AR *genes (Table 3).

Table 4 shows the frequency of parental consanguinity, familial recurrence, and severity of ambiguous genitalia in the three groups. The frequency of parental consanguinity was significantly higher in patients with 5*α*-reductase type 2 deficiency (*χ* ^2^_(2)_ = 19.86, *P* = 0.00005), whereas familial recurrence was significantly more frequent in the groups of PAIS (*χ* ^2^_(2)_ = 8.14, *P* = 0.02). The severity of ambiguous genitalia according to Sinnecker et al. [9] classification (*χ* ^2^_(2)_ = 15.49, *P* = 0.0004) and according to Ahmed et al. [10] score (*χ* ^2^_(2)_ = 20.89, *P* = 0.00003) was significantly higher in the group of 5*α*-reductase type 2 deficiency and PAIS in relation to undetermined cases. Analyzing only the patients with 5*α*-reductase type 2 deficiency and PAIS, the Sinnecker et al. [9] classification was significantly higher in PAIS (*χ* ^2^_(2)_ = 6.41, *P* = 0.04), while the Ahmed et al. [10] score did not show significant differences between these two groups of patients (Fisher = 1.00). However, these classifications showed high negative correlation (*r* = −0.675, *P* = 0.0001).

Table 5 shows range, mean and standard deviation of age at first evaluation, weight and height at birth, penile length (in z score), levels of T, Δ4-androstenedione, and T/Δ4 and T/DHT ratios in the three groups. The levels of LH and FSH were not compared because only a few patients were in pubertal age in each group of diagnosis (5*α*-reductase type 2 deficiency: 4 patients, PAIS: 1 patient, and undetermined: 2 patients). The age at first evaluation was significantly lower in idiopathic cases (*P* = 0.02). Weight (*P* = 0.002) and length (*P* = 0.02) at birth and T/DHT ratio (*P* = 0.0001) were significantly lower in undetermined cases, and penile length was significantly higher in this group (*P* = 0.0001). The T (*P* = 0.07) and Δ4-androstendione (*P* = 0.12) levels and T/Δ4 ratio (*P* = 0.32) did not differ among the three groups. All patients showed normal Δ4-androstendione levels for age and pubertal stage and T/Δ4 ratio above 0.8. 

## 4. Discussion

Clinical and laboratorial investigation must include careful and precise anatomical and hormonal studies (both basal and after stimulation) prior to gender assignment in 46,XY patients with undermasculinization (46,XY DSD), which can be difficult in most cases [1, 8, 12]. The phenotypes of 46,XY DSD due to 5*α*-reductase type 2 deficiency, PAIS, and disorders in testosterone synthesis may be indistinguishable in newborns [1–6, 8, 12]. The differential diagnosis of PAIS and 5*α*-reductase type 2 deficiency should be established as soon as possible because individuals with PAIS are usually recommended to be raised as females, whereas those with 5*α*-reductase type 2 deficiency as males, when the diagnosis is made early in childhood [1, 6, 8, 12–14].

A correct and early diagnosis is very important because as a result of pre- and/or postnatal brain exposure to androgens, almost 70% of individuals with 5*α*-reductase type 2 deficiency and 46,XY karyotype raised as girls develop a male gender identity and change the gender behavior in adolescence or early adulthood [2, 4, 15–18]. The degree of external genital masculinization at birth does not seem to be related to gender role changes [18].

At puberty, the differential diagnosis of PAIS and 5*α*-reductase type 2 deficiency can be easier due to the presence of gynecomastia, little genital virilization, and body hair in patients with PAIS, whereas in patients with 5*α*-reductase type 2 deficiency, there is genital virilization, although not always with adequate penile growth, absence of gynecomastia, and absent or hypoplastic prostate [2–5]. Also at puberty, serum levels of LH and T are abnormally elevated in patients with PAIS [10]. However, in prepubertal patients with PAIS, serum concentrations of T and LH are generally normal and do not help to establish the diagnoses [13]. In addition, T/DHT is usually elevated in prepubertal patients with 5*α*-reductase type 2 deficiency [4]. Surprisingly, in this sample, the T/DHT ratio did not allow differentiation between patients with PAIS and 5*α*-reductase type 2 deficiency, but it was important to differentiate these two groups from undetermined cases. Probably, this result was due to the sensitivity of laboratory methods. Unfortunately, we could not confirmed these results using more specific methods like liquid chromatography linked with tanden mass spectrometry (LC-MS/MS) or immunoassays after organic solvent extraction [19]. Δ4-androstenedione and T/Δ4 ratios were normal in all patients evaluated. According to George et al. [20], the diagnosis of 17*β*-hydroxysteroid dehydrogenase III deficiency in infants younger than 6 months can be excluded with a basal T/Δ4 ratio above 0.8, with a sensitivity of 100% and in prepubertal children with the same value of T/Δ4 ratio after hCG stimulation test, with a sensitivity of 90%. 

In the present study, among 51 families evaluated, 7 (13.7%) showed homozygous or compound heterozygous mutations in *SRD5A2* gene, confirming the diagnosis of 5*α*-reductase type 2 deficiency, 5 (9.8%) showed hemizygous mutation in *AR* gene, confirming the diagnosis of PAIS, and the remaining 39 (76.4%) cases showed normal molecular analysis of *SRD5A2 *and *AR *genes. Based on the literature available, we expected more cases of PAIS [21, 22] and up to 30–40% of undetermined cases [6, 8]. In addition, probably more patients would be diagnosed with PAIS and mutations in the *AR* gene if the *AR* promoter region and 3′UTR were evaluated, and we must also remember that the *AR* gene in target tissues from patients with hypospadias is more methylated than in control children, resulting in a decreased expression of the *AR*. This epigenetic alteration of the *AR* gene might be involved in the pathogenesis of hypospadias [23].

As expected, the frequency of parental consanguinity was higher in patients with 5*α*-reductase type 2 deficiency, which is a male-limited autosomal recessive disorder (OMIM 264600), whereas the familial recurrence was higher in PAIS, an X-linked disorder (OMIM 300068).

Weight and length at birth were lower in undetermined cases, suggesting that metabolic and endocrine disorders as fetal malnutrition could play a role in poor external genitalia development. Morel et al. showed that, in comparison with PAIS, undetermined 46,XY DSD was characterized by a high incidence of prematurity and/or intrauterine growth retardation (30%) [8]. In the present study, 12/39 (30.7%) undetermined cases had birth weight lower than 2,500 g. De Andrade Machado Neto et al. [24] showed an association between prenatal growth retardation and 46,XY DSD which may be due to genetic factors not clarified yet or to environmental factors which act early in gestation. Scaramuzzo et al. recently demonstrated that in the first days of life, small-for-gestational-age male pre-term newborns have reduced testosterone levels compared with adequate-for-gestational-age preterm newborns, independently from the presence of abnormalities of the external genitalia [25]. Low testosterone levels were not observed in our patients with low birth weight and length probably due to the gestational age, as all patients included in this study were born at term. Nutrition is the major intrauterine environmental factor that alters expression of the fetal genome and may have lifelong consequences (fetal programming). Alterations in fetal nutrition and endocrine status may result in developmental adaptations that permanently change the structure, physiology, and metabolism of the offspring, predisposing individuals to metabolic, endocrinological, and cardiovascular diseases in adult life [26, 27]. Furthermore, it may be that sexual plasticity during development explains the vulnerability of organisms to androgen influences, such as environmental oestrogens or endocrine disruptors [28–31]. The severity of ambiguous genitalia was higher in 5*α*-reductase type 2 deficiency in comparison with PAIS and undetermined cases. The 5*α*-reductase type 2 deficiency has frequently a classical syndrome of pseudovaginal perineoscrotal hypospadias, characterized by a predominantly female phenotype at birth and significant virilization at puberty [9]. Recent reports have shown the clinical spectrum to be heterogeneous, ranging from the classic phenotype to males with hypospadias and even micropenis [32].

The age at first evaluation was lower in undetermined cases, and this data can be associated with severity of ambiguous genitalia: 5*α*-reductase type 2 deficiency and PAIS groups have more cases with Sinnecker et al. [9], classification grades 3 and 4, and Ahmed et al. [10] score below 3.5 than undetermined cases and may be more frequently underdiagnosed. The penile length, that was higher in undetermined cases, reinforces this hypothesis.

In conclusion, birth weight and length, parental consanguinity, familial recurrence, severity of ambiguous genitalia, penile length, and T/DHT ratio may help the investigation of 46,XY patients with ambiguous genitalia and normal testosterone synthesis.

## Figures and Tables

**Table 1:** Data from 9 patients (7 families) of 5*α*-reductase type 2 deficiency.

Case	Age 1 (yr)	Birth weight (g)	Birth length (cm)	Penile (z)	Genital^1^	Genital^2^	T (ng/mL)	T/DHT	Mut1	Mut2
1^c^	0.06	3220	50	−4.6	4	3.0	2.8	28	p.G183S	p.G183S
2^c^	18.2	2900	48	−4.2	3	2.5	9.0	45	c.418delT	c.418delT
3^c^	14.6	2800	47	−4.1	3	2.0	2.8	28	p.R246W	p.R246W
4	5.4	2700	48	−4.1	2	9.0	2.2	73	c.278delG	c.278delG
5^∗1^	3.0	2810	47	−4.0	3	1.0	1.8	60	p.Q126R	p.G158R
6^∗1^	0.05	3500	50	−4.0	3	3.0	2.2	44	p.Q126R	p.G158R
7^c,r^	16.7	2600	47	−4.2	3	2.5	4.9	82	p.G196S	p.G196S
8^∗2c,r^	17.3	2900	49	−3.9	3	5.5	13.6	68	p.Q126R	p.Q126R
9^∗2c,r^	11.0	2600	48	−3.1	3	4.0	1.9	63	p.Q126R	p.Q126R

Age1: age at first evaluation, T: total testosterone, Mut1: mutation 1, Mut2: mutation 2, +: present, −: absent, *: related, c: presence of consanguinity, r: presence of familial recurrence, Genital^1^: external genitalia according to Sinnecker et al. [9], Genital^2^: external genitalia according to Ahmed et al. [10].

**Table 2:** Data from 10 patients (5 families) of PAIS.

Case	Age1 (yr)	Birth weight (g)	Birth length (cm)	Penile (z)	Genital^1^	Genital^2^	T (ng/mL)	T/DHT	Mutation
1^∗1 c^	28.8	2900	48	−8.7	4	1.0	10.0	33	p.L830F
2^∗1 c^	18.8	3330	48	−8.4	4	2.0	15.0	50	p.L830F
3^∗1 c^	3.0	2800	47	−6.2	4	2.0	3.2	32	p.L830F
4^∗1 c^	1.6	2650	46	−5.2	4	2.0	1.8	60	p.L830F
5^∗1 c^	0.2	3180	50	−6.2	4	5.5	2.0	67	p.L830F
6^∗2^	0.8	2630	46	−2.7	2	5.5	1.9	63	p.A596T
7^∗2^	0.2	2900	46	−7.5	2	2.0	1.7	34	p.A596T
8	1.3	3950	49	−3.1	2	6.0	2.9	72	p.A896V
9	19.2	3400	51	−7.5	3	1.0	9.6	46	p.R855H
10^c^	6.1	3150	49	−4.1	3	3.0	2.3	57	p.M742V

Age1: age at first evaluation, T: total testosterone, +: present, −: absent, *: related, c: presence of consanguinity, r: presence of familial recurrence, Genital^1^: external genitalia according to Sinnecker et al. [9], Genital^2^: external genitalia according to Ahmed et al. [10].

**Table 3:** Data from 39 patients without molecular defects in *SRD5A2 *and *AR* genes.

Case	Age1	Birth weight (g)	Birth length (cm)	Penile (z)	Genital^1^	Genital^2^	T (ng/mL)	T/DHT
1	0.6	2800	47	−4.0	3	5.5	3.7	18
2^r^	0.08	2650	46	−2.7	2	6.0	3.5	12
3	0.04	2740	47	−4.0	2	6.0	1.9	95
4	0.4	2410	46	−2.7	2	5.0	1.7	8
5	2.8	2700	48	−1.2	2	10.0	1.7	8
6^r^	4.1	2800	47	−1.1	2	10.0	2.1	7
7	0.6	1650	41	−3.4	2	6.5	2.1	10
8	0.8	2580	46	−3.0	2	8.0	2.7	13
9	13.8	2900	47	−6.5	3	5.0	1.7	1
10^r^	20.7	2700	46	−5.6	2	6.0	16.0	5
11	0.8	3000	47	−3.4	3	6.0	1.5	3
12	2.4	2500	49	−3.1	3	1.0	1.9	4
13^r^	2.3	2010	44	−3.7	2	5.0	1.8	6
14^r^	0.8	2900	47	−3.0	2	6.0	3.4	11
15	8.1	2800	48	−2.0	2	10.0	1.8	30
16^r^	0.3	2650	47	−2.7	2	5.5	3.6	18
17	10.2	2110	43	−3.1	2	8.0	1.8	18
18	3.8	1430	39	−4.0	2	5.5	2.0	22
19^r^	0.5	2500	45	−2.7	2	6.0	1.9	9
20	0.04	2810	46	−2.1	3	6.0	2.4	6
21	0.1	2160	43	−2.7	2	6.0	2.3	8
22	0.2	2600	47	−3.4	2	5.0	3.4	8
23^r^	0.1	3000	48	−4.2	2	5.0	2.0	10
24^r^	0.5	2700	48	−2.4	2	9.0	6.6	17
25	0.07	1740	42	−3.1	2	5.0	1.8	9
26^c^	0.4	1700	41	−2.1	2	9.0	2.7	9
27	1.9	2330	42	−2.9	2	6.0	3.4	17
28	0.02	2900	49	−2.7	2	6.0	1.8	9
29	0.2	1540	40	−4.5	2	6.0	1.7	8
30	0.06	2190	42	−3.4	3	3.0	2.6	5
31	0.7	1430	40	−3.4	2	6.0	1.6	8
32	0.2	2250	43	−2.4	3	5.5	1.6	55
33^r^	0.3	2990	49	−2.4	2	6.0	1.6	16
34	10.9	2800	47	−6.6	3	1.0	1.7	8
35	2.6	2700	49	−3.1	2	6.0	4.0	13
36	0.1	2800	50	−2.1	2	8.5	1.9	18
37^r^	3.7	2450	49	−2.7	2	4.0	2.5	12
38^c,r^	0.02	2900	48	−2.7	3	6.0	2.2	4
39^r^	2.7	3400	51	−5.9	2	1.0	1.9	12

Age1: age at first evaluation, +: present, −: absent, c: presence of consanguinity, r: presence of familial recurrence, T: total testosterone, Genital^1^: external genitalia according to Sinnecker et al. [9], Genital^2^: external genitalia according to Ahmed et al. [10].

**Table 4:** Data from 7 families with 5*α*-reductase type 2 deficiency, 5 families with PAIS, and 39 isolated undetermined cases.

		5*α*-reductase type 2 deficiency	PAIS	Undetermined	*P* value*
Parental consanguinity	+	5	2	2	*P* = 0.00005
	−	2	3	37	
Familial recurrence	+	3	5	13	*P* = 0.02
	−	4	0	26	
Ambiguous genitalia Sinnecker et al. [9]	**2**	1	3	29	*P* = 0.0004
	**3**	7	2	10	
	**4**	1	5	0	
Ambiguous genitalia Ahmed et al. [10]	**<3.5**	6	7	4	*P* = 0.00003
	**≥3.5**	3	3	35	

*Chi-square test.

**Table 5:** Data from 9 patients with 5*α*-reductase type 2 deficiency, 10 patients with PAIS, and 39 isolated undetermined cases.

	5*α*-reductase type 2 deficiency	PAIS	Undetermined	*P* value*
Age at first consultation (yr)	9.6 ± 7.5 (0.05–18.2)	8.0 ± 10.0 (0.2–28.8)	2.5 ± 4.5 (0,02–20,7)	*P* = 0.02
Birth weight (g)	2890 ± 290 (2600–3500)	3100 ± 400 (2630–3950)	2470 ± 460 (1700–3000)	*P* = 0.002
Birth length (cm)	48 ± 1 (47–50)	48 ± 2 (46–51)	46 ± 3 (39–50)	*P* = 0.02
Penile length (z)	−4.0 ± 0.4 (−4.6–−3.1)	−6.0 ± 2.1 (−8.7–−2.7)	−3.2 ± 1.2 (−6.6–−1.1)	*P* = 0.0001
T (ng/mL)	4.6 ± 4.1 (1.8–13.6)	4.5 ± 4.4 (1.7–15.0)	2.6 ± 1.4 (1.5–9.1)	*P* = 0.07
Δ4 (ng/mL)	1.4 ± 0.4 (0.8–1.9)	1.1 ± 0.4 (0.7–1.9)	1.2 ± 0.3 (0.7–1.7)	*P* = 0.12
T/Δ4 ratio	3.0 ± 1.9 (1.5–7.2)	3.6 ± 1.9 (2.1–7.9)	2.7 ± 0.9 (1.8–7.0)	*P* = 0.32
T/DHT ratio	54 ± 19 (28–82)	51 ± 15 (32–72)	14 ± 16 (1–95)	*P* = 0.0001

*Kruskal-Wallis test.
